# Routinediagnostik vor ohrchirurgischen Eingriffen – Ergebnisse einer Umfrage unter deutschsprachigen HNO-Kliniken

**DOI:** 10.1007/s00106-025-01556-w

**Published:** 2025-04-09

**Authors:** Joseph Morgenstern, Marcus Neudert, Thomas Zahnert, Susen Lailach

**Affiliations:** Klinik und Poliklinik für Hals-Nasen-Ohren-Heilkunde, Kopf- und Halschirurgie, Universitätsklinikum „Carl-Gustav-Carus“ an der Technischen Universität Dresden, Fetscherstraße 74, Haus 5, 01307 Dresden, Deutschland

**Keywords:** Audiometrie, Ohrchirurgie, Standardisierung, Leitlinien, Zielparameter, Audiometry, Ear surgery, Standardization, Guidelines, Outcome parameters

## Abstract

**Hintergrund:**

Die Diagnostik vor ohrchirurgischen Eingriffen ist wichtiger Bestandteil der ohrenärztlichen Tätigkeit in Kliniken. Bislang existiert national und international noch kein Diagnostikstandard. Vielmehr sind die genutzten Testbatterien abhängig von klinikeigenen Lehrmeinungen.

**Fragestellung:**

Ziel der Arbeit war es, durch eine Umfrage im deutschsprachigen Raum die Routinediagnostikmethoden vor verschiedenen ohrchirurgischen Eingriffen zu erfassen.

**Methoden:**

Es wurde ein Online-Fragebogen mit 18 allgemeinen Fragen zur Ausstattung der Klinik und Verfügbarkeit von Tests sowie je sechs Fragen zu spezifischen Ohroperationen an die Klinikleiter/-innen versendet.

**Ergebnisse:**

Von 180 angeschriebenen Kliniken nahmen 56 Kliniken (31 %) an der Umfrage teil. Für acht spezifische Operationen (sanierende Ohr-Operation mit und ohne Cholesteatom, hörverbessernde Operation, Stapesplastik, Gehörgangsstenosen-Operation, Gehörgangsatresie-Operation, Implantation von aktiven Mittelohrimplantaten und Cochleaimplantat-Operationen) konnten die im deutschsprachigen Raum mehrheitlich genutzten Testbatterien bestimmt werden. Es zeigte sich, dass bei klar definierten Diagnostikbatterien diese mehrheitlich in den klinikeigenen Diagnostikschritten implementiert werden. Eine apparative neurootologische Diagnostik erfolgt im deutschsprachigen Raum vorzugsweise vor einer Hörimplantatversorgung, hat jedoch bei mittelohrchirurgischen Eingriffen in den deutschsprachigen Kliniken nur einen geringen Stellenwert.

**Schlussfolgerung:**

Im deutschsprachigen Raum zeigt sich eine Inhomogenität der Diagnostikpfade vor ohrchirurgischen Eingriffen. Die Ergebnisse dieser Umfrage liefern eine weitere Diskussionsgrundlage zur Erarbeitung eines Diagnostikstandards, welcher in entsprechenden Leitlinien und Konsensuspapieren zu erarbeiten ist.

## Abstract (en)

**Background:**

Diagnostic testing prior to ear surgery is an important part of the work of ear specialists in hospitals. To date, there is no national or international diagnostic standard. Rather, the test batteries used are dependent on hospitals’ own doctrinal opinions.

**Objective:**

The aim of this study was to conduct a survey in German-speaking countries to determine the routine diagnostic methods used prior to various ear surgery procedures.

**Methods:**

An online questionnaire with 18 general questions on the hospital’s equipment and availability of tests and 6 questions on specific ear operations was sent to the departmental directors.

**Results:**

Of the 180 hospitals contacted, 56 (31%) took part in the survey. For eight specific operations (rehabilitative ear surgery with and without cholesteatoma, hearing-improving surgery, stapesplasty, ear canal stenosis surgery, ear canal atresia surgery, implantation of active middle ear implants, and cochlear implant surgery), the test batteries most commonly used in German-speaking countries could be determined. It was found that the majority of clearly defined diagnostic batteries are implemented in the hospital’s own diagnostic steps. In German-speaking countries, neurootological diagnostics are preferably carried out before treatment with hearing implants, but are of only minor importance for middle ear surgery in German-speaking hospitals.

**Conclusion:**

There is inhomogeneity regarding diagnostic pathways in ear surgery in German-speaking countries. The results of this survey provide a further basis for discussion concerning the development of a diagnostic standard, which is to be developed in corresponding guidelines and consensus papers.

## Hintergrund

Die Diagnostik vor ohrchirurgischen Eingriffen und deren Erfolgsbewertung sind seit jeher integraler Bestandteil der Hals-Nasen-Ohren(HNO)-ärztlichen Tätigkeit in Kliniken. Die Diagnostikpfade sowie die Behandlungsstrategien sind dabei abhängig von der klinikeigenen Lehrmeinung.

Bereits seit Anfang der 1990er-Jahre existieren Bestrebungen, die Berichtserstattung audiologischer Ergebnisse in klinischen Studien zu standardisieren [6, 24, 25]. Die Qualitätsanalyse der bisherigen Studienlage sowie Umfragen zur audiologischen Praxis konnten jedoch deutliche Defizite hinsichtlich der Vollständigkeit der Dokumentation, jedoch auch die Heterogenität der Diagnostikmethoden und Zielparameter aufzeigen [21, 24, 27, 32]. Da für klinische Studienfragestellungen oft auf die Ergebnisse der klinischen Routinediagnostik zurückgegriffen wird, lässt sich auch für den klinischen Alltag eine Heterogenität der Diagnostikmethoden absehen. Hierbei sind nicht nur Unterschiede in der Auswahl der entsprechenden Untersuchungsmethoden, sondern auch in deren Durchführung zu erwarten. Bisherige Untersuchungen konnten beispielsweise für die Durchführung einer Sprachaudiometrie klinikübergreifend deutliche Unterschiede hinsichtlich Sprachmaterial und Sprachschallpegel aufzeigen [24, 25, 27].

### Berichtsstandards

International sind erste Versuche zur Bereitstellung eines Berichtsstandards von Ergebnissen ohrchirurgischer Eingriffe publiziert [6, 17, 37]. Die Mängel der durch die amerikanische Fachgesellschaft forcierten „Standards“ sind hinlänglich bekannt und diskutiert [21, 27, 28]. Zur internationalen Vereinheitlichung von prä- und postoperativen Testverfahren im klinischen Alltag existieren bislang noch keine Empfehlungen. Auch innerhalb der deutschsprachigen Fachgesellschaften ist dieses Defizit bekannt. Insbesondere in der Arbeitsgemeinschaft Deutschsprachiger Audiologen, Otologen und Neurootologen (ADANO) wird seit Jahren die Einigung auf eine präoperative Testbatterie in das Zentrum der Diskussion gerückt. Doch selbst den verfügbaren deutschsprachigen Leitlinien (Tab. 1) mangelt es an einer Konkretisierung der prä- und postoperativen Diagnostik [9–11]. Für einzelne Krankheitsbilder bzw. ohrchirurgische Eingriffe, u. a. Otosklerose, sind Leitlinien gar nicht erst verfügbar. Allein in der zuletzt publizierten Leitlinie zur Cochleaimplantatversorgung wurde nach vielfältiger Diskussion eine Vielzahl prä- und postoperativer Testverfahren konkretisiert und in einem Register zur Erfassung der Versorgungsqualität integriert [12].

**Tab. 1:** Festlegung präoperativer Diagnostik in den aktuellen Leitlinien (Stand 11/2023)

Leitlinie	Audiologische Diagnostik	Neurootologische Diagnostik	Bildgebung	PROM
*„Cholesteatom“* S1-Leitlinie AWMF-Register-Nr. 017/006 Stand 13.06.2014 Gültig bis 12.06.2019^a^	Stimmgabelversuch Reintonaudiometrie	Frenzel-Brille Überprüfung Fistelsymptom	Im Einzelfall (Röntgen-Schüller, CT, MRT)	Nicht spezifiziert
*„Chronisch mesotympanale Otitis media (CMOM)“* S2k-Leitlinie AWMF-Register-Nr. 017/074 Stand 20.09.2020 Gültig bis 19.09.2025	Stimmgabelversuch Reintonaudiometrie Sprachaudiometrie (fakultativ)	Nicht spezifiziert	Fakultativ: Röntgen-Schüller, CT, DVT	Nicht spezifiziert
*„Implantierbare Hörgeräte“* S2k-Leitlinie AWMF-Register-Nr. 017/073 Stand 31.12.2017 Gültig bis 30.12.2022	Reintonaudiometrie Sprachaudiometrie in Ruhe (ggf. auch im Störschall) Otoakustische Emissionen BERA Hörgeräteüberprüfung	Nicht spezifiziert	CT und MRT	Ggf. Einsatz von Frageninventaren (APHAB, HHIE; IOI-HA, SSQ, BBSS)
*„Cochlea-Implantat Versorgung“* S2k-Leitlinie AWMF-Register-Nr. 017/071 Stand 31.10.2020 Gültig bis 30.10.2025	Reintonaudiometrie Sprachaudiometrie in Ruhe Sprachaudiometrie im Störgeräusch Hörgeräteüberprüfung Tympanometrie Stapediusreflexe Otoakustische Emissionen BERA ggf. ECochG, E‑BERA, ASSR	Kalorik und/oder vKIT ggf. VEMP	CT oder DVT, MRT	Ggf. Einsatz von NCIQ, SSQ, HHIE

*PROM* Patient-Reported Outcome Measures, *CT* Computertomographie, *MRT* Magnetresonanztomographie, *DVT* digitale Volumentomographie, *BERA* Brainstem-Evoked Response Audiometry, *ASSR* Auditory Steady-State Response, *ECochG* Elektrocochleographie, *vKIT* Video-Kopfimpulstest, *VEMP* vestibulär evozierte myogene Potenziale, *NCIQ* Nijmegen Cochlear Implant Questionnaire, *HHIE* Hearing Handicap Inventory for the Elderly, *SSQ* Speech Spatial and Qualities of Hearing Scale, *APHAB* Abbreviated Profile of Hearing Aid Benefit, *BBSS* Bern Benefit in Single-Sided Deafness Questionnaire, *IOI-HA* International Outcome Inventory for Hearing Aids

^a^ Klassenupgrade S2k-Leitlinie am 01.09.2023 angemeldet

Eine qualitativ hochwertig zusammengestellte Testbatterie spiegelt sich dabei nicht in der Quantität der Messverfahren wider. Vielmehr gilt es an dieser Stelle, möglichst evidenzbasiert Testverfahren zu selektieren, welche zur Beschreibung des Funktionszustands des Sinnesorgans Ohr und zur Sicherstellung der Diagnose bzw. Operationsindikation, Erfolgsprognose und Ergebnisbeschreibung von Nöten sind. Neben dem eigenen Qualitätsanspruch beeinflussen medikolegale Aspekte und ökonomische Randbedingungen die Auswahl geeigneter Testbatterien.

### Medikolegalität

Der Begriff „medikolegale Aspekte“ umfasst verschiedene Themen, bei denen medizinisches Wissen und Fachkenntnisse in einem rechtlichen Kontext angewendet werden. Dazu gehören Aspekte wie ärztliche Behandlungsfehler, die Einwilligung in die Behandlung, die ärztliche Schweigepflicht, die medizinische Dokumentation und die Rolle von Sachverständigen im Gesundheitswesen als Zeugen in Gerichtsverfahren [22, 29, 35]. Präoperative Diagnostik dient hierbei zur Unterstützung der Diagnosefindung und bildet damit eine Grundlage für Therapieplanung und informierte Einwilligung. Zudem ist sie als Ausgangswert für Erfolgskontrolle und Monitoring von Komplikationen erforderlich. Nach § 630f BGB besteht für Behandelnde eine Dokumentationspflicht [4]. Hierbei hat das ärztliche Handeln gemäß § 630a Abs. 2 BGB nach anerkannten fachlichen Standards zu erfolgen. Diagnosefehler, z. B. durch Nichterheben elementarer Befunde oder fehlende Abklärung von Verdachtsdiagnosen, sind eine Unterform der Behandlungsfehler [4].

Der „Behandlungsstandard“ bezieht sich auf das Maß an Behandlung, das ein kompetenter und gewissenhafter Arzt in einem bestimmten Bereich oder Fachgebiet unter ähnlichen Umständen leisten würde. Der Standard basiert auf medizinisch-wissenschaftlichen Kenntnissen und/oder praktischen/ärztlichen Erfahrungen, welche von der medizinischen Fachwelt allgemein als angemessen und wirksam anerkannt sind [5]. Der Behandlungsstandard ist kein starres Regelwerk, sondern vielmehr ein flexibles Konzept, das Faktoren wie den Zustand der Patient/-innen, die verfügbaren Ressourcen und den medizinischen und technischen Fortschritt berücksichtigt. Der Behandlungsstandard bezeichnet also nicht ein denkbares Maximum oder Optimum an möglicher Leistung, sondern einen Sorgfaltsmaßstab [15]. Die Diagnostik vor ohrchirurgischen Eingriffen erfordert demnach eine Testbatterie, welche eine sorgfältige Diagnosestellung, Therapie und Beratung der Patient/-innen ermöglicht.

### Fragestellung

Diese Arbeit soll den aktuellen Stand zur Verbreitung und Akzeptanz von Diagnostikverfahren vor ohrchirurgischen Eingriffen innerhalb deutschsprachiger HNO-Kliniken erfassen und hierbei eine Grundlage zur möglichst standortunabhängigen Vereinheitlichung der diagnostischen Testbatterien liefern.

## Studiendesign und Untersuchungsmethoden

Die Befragung erfolgte über einen 15-seitigen anonymen Online-Fragebogen (SoSci Survey, SoSci Survey GmbH, München, Deutschland) mit 18 allgemeinen Fragen zur Ausstattung der Klinik und Verfügbarkeit von Tests sowie je sechs Fragen zu spezifischen Operationen (sanierende Ohr-Operation mit und ohne Cholesteatom, hörverbessernde Operation, Stapesplastik, Gehörgangsstenosen-Operation, Gehörgangsatresie-Operation, Implantation von aktiven Mittelohrimplantaten und Cochleaimplantat-Operationen [CI-Operationen]). Es handelte sich dabei um geschlossene Fragen mit Ein- und Mehrfachantwort sowie einzelnen Eingabemöglichkeiten. Der Link wurde an 180 Klinikleitungen in Deutschland, Österreich und der Schweiz geschickt, die Befragung konnte von den Klinikleiter/-innen delegiert werden. Die Kontaktdaten wurden durch die Deutsche Gesellschaft für Hals-Nasen-Ohren-Heilkunde, Kopf- und Hals-Chirurgie e. V. und die Österreichische Gesellschaft für Hals‑, Nasen‑, Ohrenheilkunde, Kopf- und Halschirurgie bereitgestellt bzw. über die Klinikliste im Internetauftritt der Schweizerischen Gesellschaft für Oto-Rhino-Laryngologie, Hals- und Gesichtschirurgie ermittelt. Die Befragung erfolgte im Zeitraum August 2022 bis Juli 2023. Der vollständige Fragebogen ist im Online-Zusatzmaterial angefügt. Die deskriptive Auswertung und die Erstellung der Abbildungen erfolgten mit Microsoft Excel 2021 (Fa. Microsoft Inc., Redmond, WA, USA) und OriginPro Version 2023b (Fa. OriginLab Corporation, Northampton, MA, USA).

## Ergebnisse

### Teilnehmende Kliniken

**Abb. 1:**
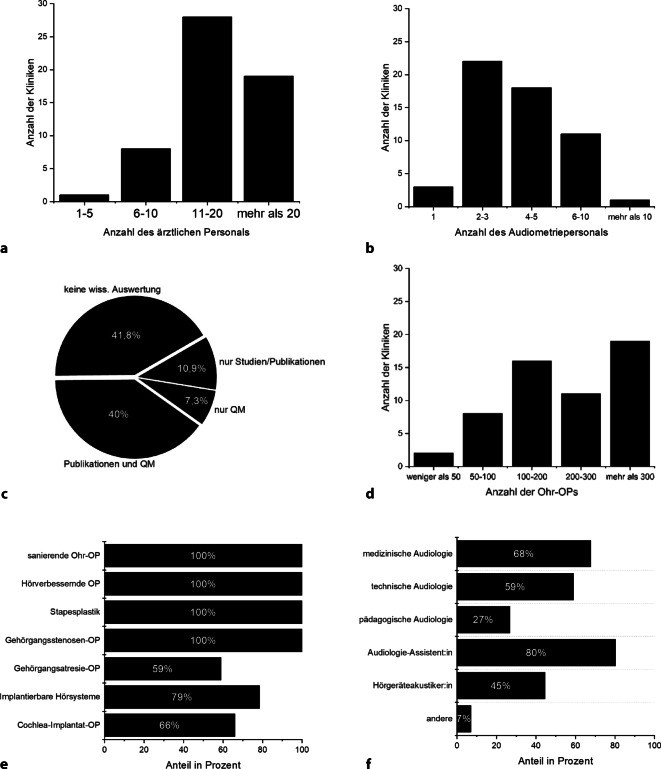
Charakteristik der an der Umfrage teilnehmenden HNO-Kliniken (*n* = 56). **a** Anzahl des ärztlichen Personals *(Angabe der absoluten Anzahl)*, **b** Anzahl der Mitarbeiter im audiologischen Funktionsbereich *(Angabe der absoluten Anzahl)*, **c** Anteil der Kliniken mit wissenschaftlicher Tätigkeit *(QM, Qualitätsmanagement)*, **d** Anzahl der jährlichen Ohroperationen *(Angabe der absoluten Anzahl)*, **e** operatives Spektrum der an der Umfrage teilnehmenden Kliniken, **f** Vorhaltung von Qualifikation der im audiologischen Bereich tätigen Mitarbeiter/-innen. Dargestellt ist jeweils der Anteil der befragten Kliniken, die Mitarbeiter/-innen mit der entsprechenden Qualifikation vorhalten

Insgesamt haben 56 Kliniken (31 % der angefragten Kliniken) an der Umfrage teilgenommen. Hierbei handelte es sich um 42 deutsche HNO-Kliniken, 12 österreichische HNO-Kliniken und zwei Schweizer HNO-Kliniken. Der Dateneingang erfolgte anonym. Die Charakteristik der einzelnen Kliniken bzw. deren angegebene personelle Ausstattung ist der Abb. 1 zu entnehmen. In mehr als der Hälfte der teilnehmenden Kliniken (54 %, *n* = 30) erfolgen mehr als 200 Ohroperationen pro Jahr. Sanierende Ohroperationen, hörverbessernde Operationen und Stapesplastik zählen zu dem operativen Portfolio aller an der Umfrage teilnehmen Kliniken. Mit 71 % (*n* = 40) verfügt die Mehrheit der eingeschlossenen Kliniken über zwei bis fünf Mitarbeiter/-innen in der audiologischen Funktionsabteilung. Technische Audiologen werden in 59 % (*n* = 33) der Kliniken vorgehalten. In 45 % (*n* = 25) der an der Befragung teilnehmenden Kliniken ist ein Datenbanksystem zur Erfassung audiologischer Daten verfügbar, 23 % (*n* = 13) der Kliniken greifen dabei auf ENT-Statistics der Fa. Innoforce (Ruggell, Liechtenstein) zurück. Die erhobenen Routinedaten werden in 54 % (*n* = 30) der an der Umfrage beteiligten Kliniken auch zur Publikation wissenschaftlicher Ergebnisse genutzt.

### Audiologische Testverfahren

Zur Bewertung der Reintonaudiometrie erfolgt in 70 % (*n* = 39) der befragten Kliniken eine Mittelwertberechnung. In den meisten dieser Kliniken (23 von 39 Kliniken, 59 %) wird der Reintonmittelwert bei den Frequenzen 0,5; 1; 2 und 4 kHz gebildet. Nur in vier dieser Kliniken (10 %) erfolgt die Mittelung bei 0,5; 1; 2 und 3 kHz. In nahezu allen Kliniken (98 %, *n* = 56) wird zur Durchführung der Sprachaudiometrie der Freiburger Sprachtest vorgehalten. Der Oldenburger Satztest wird in 64 % (*n* = 36) der befragten Kliniken routinemäßig zur Diagnostik eingesetzt. In 23 Kliniken (41 %) erfolgte die Bestimmung des Sprachverstehens bei festem Sprachschallpegel. In 19 der befragten Kliniken (34 %) wird bei einem festen Pegel von 65 dB SPL und in 16 Kliniken (29 %) zusätzlich bei 80 dB SPL standardmäßig das Sprachverstehen bestimmt. In 26 Kliniken (46 %) wird bei variablem Sprachschallpegel das Sprachverstehen ermittelt. In keiner der teilnehmenden deutschsprachigen Klinik wird die von Gurgel et al. empfohlene Messung bei 40 dB über der Sprachverständlichkeitsschwelle durchgeführt. Die Hälfte der befragten Kliniken gab an, das maximale Sprachverstehen routinemäßig zur präoperativen Diagnostik zu bestimmen.

**Abb. 2:**
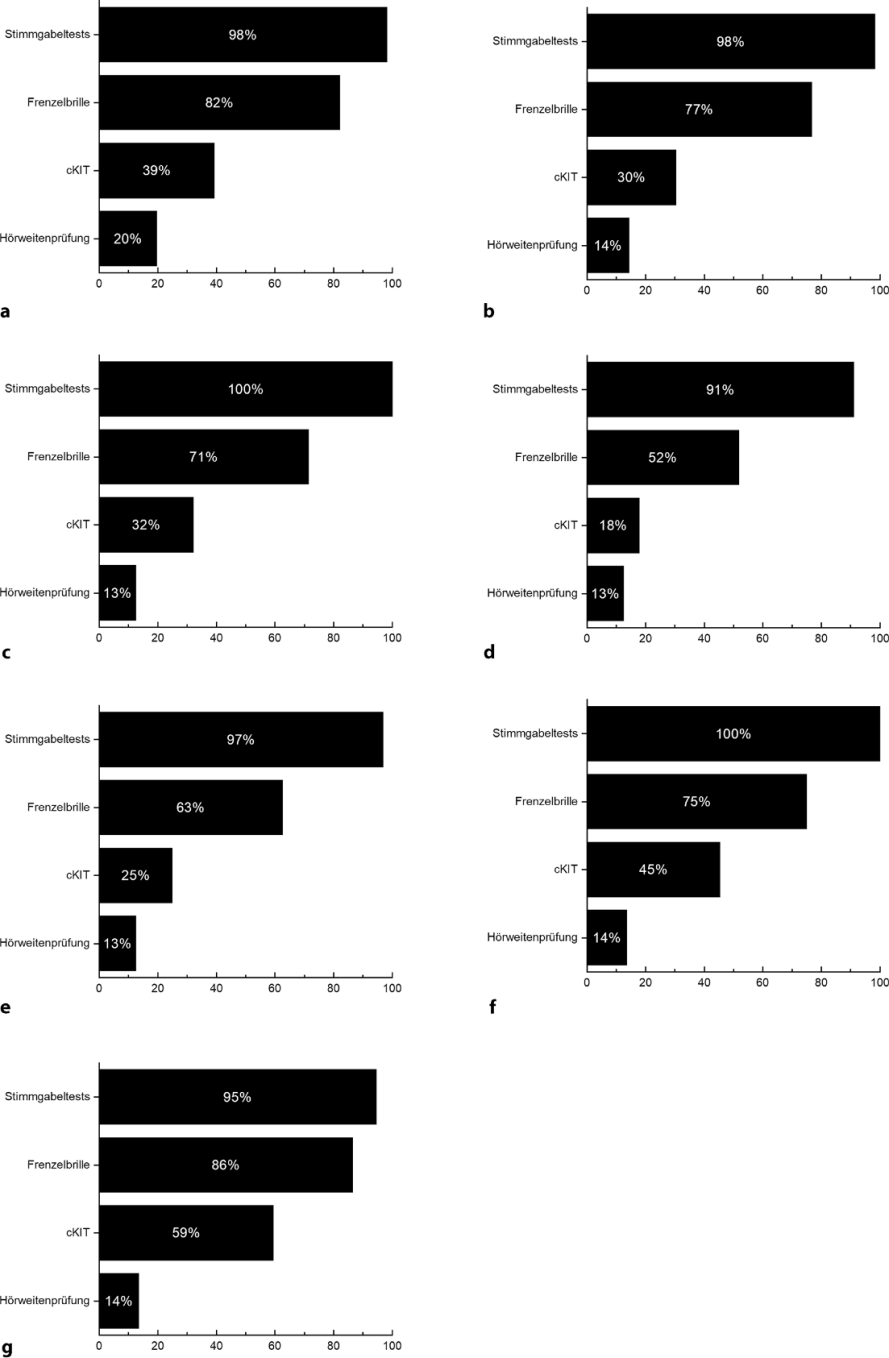
Klinische Routinediagnostik vor ohrchirurgischen Eingriffen. **a** Sanierende Ohroperationen (*n* = 56), **b** hörverbessernde Operationen (*n* = 56), **c** Stapesplastik (*n* = 56), **d** Gehörgangsstenosen-Operation (*n* = 56), **e** Gehörgangsatresie-Operationen (*n* = 32), **f** implantierbare Hörsysteme (*n* = 44), **g** CI-Operation (*n* = 38). Dargestellt ist jeweils der Anteil der befragten Kliniken, die das jeweilige Diagnostikinstrument routinemäßig vor dem spezifizierten Eingriff einsetzt. *cKIT* klinischer Kopfimpulstest

**Abb. 3:**
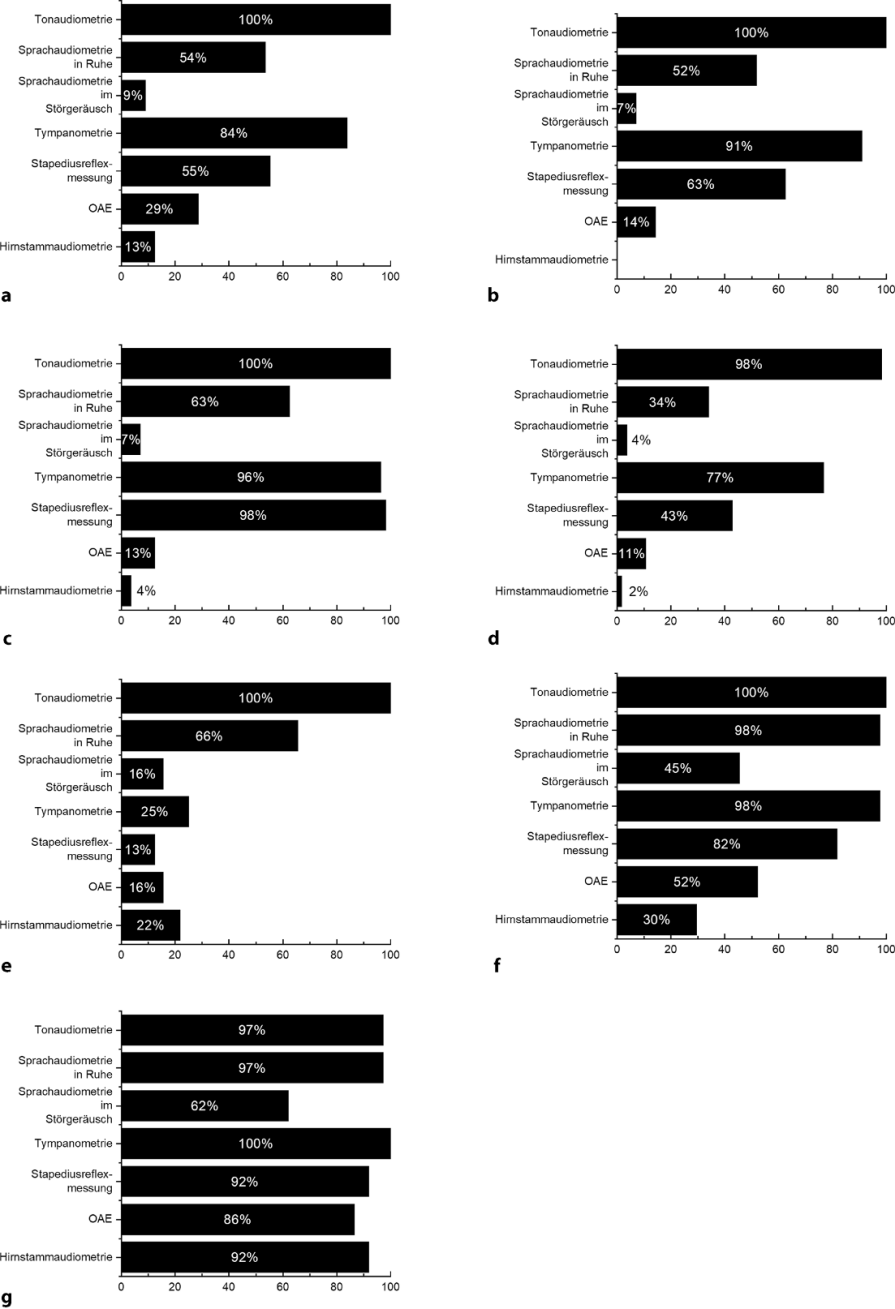
Audiologische Routinediagnostik vor ohrchirurgischen Eingriffen. **a** Sanierende Ohroperationen (*n* = 56), **b** hörverbessernde Operationen (*n* = 56), **c** Stapesplastik (*n* = 56), **d** Gehörgangsstenosen-Operation (*n* = 56), **e** Gehörgangsatresie-Operationen (*n* = 32), **f** implantierbare Hörsysteme (*n* = 44), **g** CI-Operation (*n* = 38). Dargestellt ist jeweils der Anteil der befragten Kliniken, die das jeweilige Diagnostikinstrument routinemäßig vor dem spezifizierten Eingriff einsetzt. *OAE* otoakustische Emissionen

Neben den im Folgenden aufgeführten apparativen Untersuchungen (Abb. 2, 3, 4 und 5) gehören als klinische Funktionsuntersuchungen ein Stimmgabeltest und die Untersuchung mittels Frenzel-Brille zu den routinemäßig durchgeführten Diagnostikschritten in der Mehrzahl der befragten Kliniken.

**Abb. 4:**
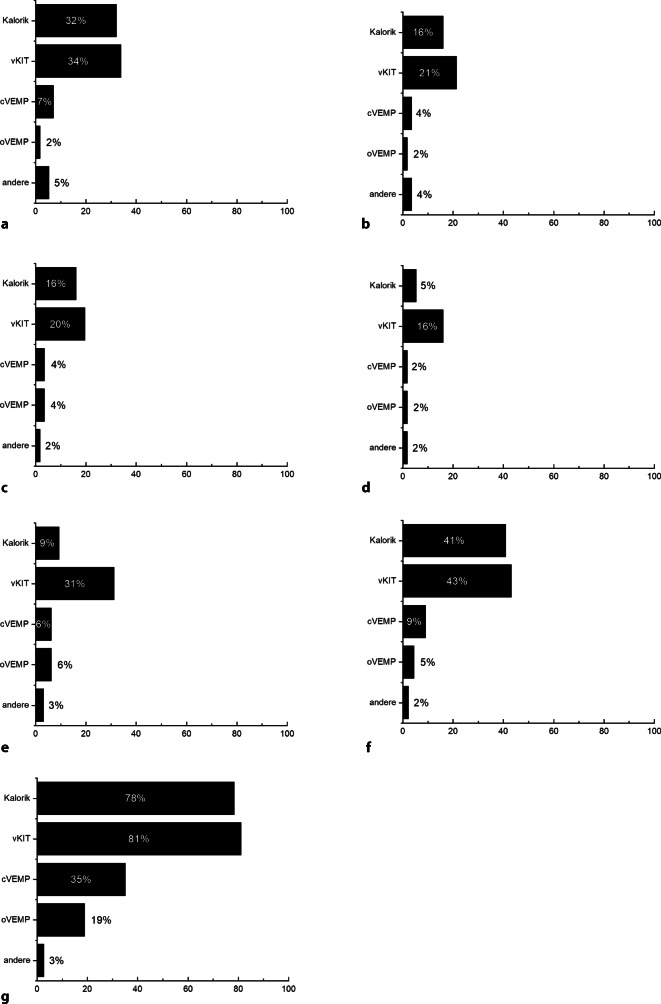
Neurootologische Routinediagnostik vor ohrchirurgischen Eingriffen. **a** Sanierende Ohroperationen (*n* = 56), **b** hörverbessernde Operationen (*n* = 56), **c** Stapesplastik (*n* = 56), **d** Gehörgangsstenosen-Operation (*n* = 56), **e** Gehörgangsatresie-Operationen (*n* = 32), **f** implantierbare Hörsysteme (*n* = 44), **g** CI-Operation (*n* = 38). Dargestellt ist jeweils der Anteil der befragten Kliniken, die das jeweilige Diagnostikinstrument routinemäßig vor dem spezifizierten Eingriff einsetzt. *vKIT* Video-Kopfimpulstest, *cVEMP* zervikale vestibulär evozierte myogene Potenziale, *oVEMP* okuläre vestibulär evozierte myogene Potenziale

**Abb. 5:**
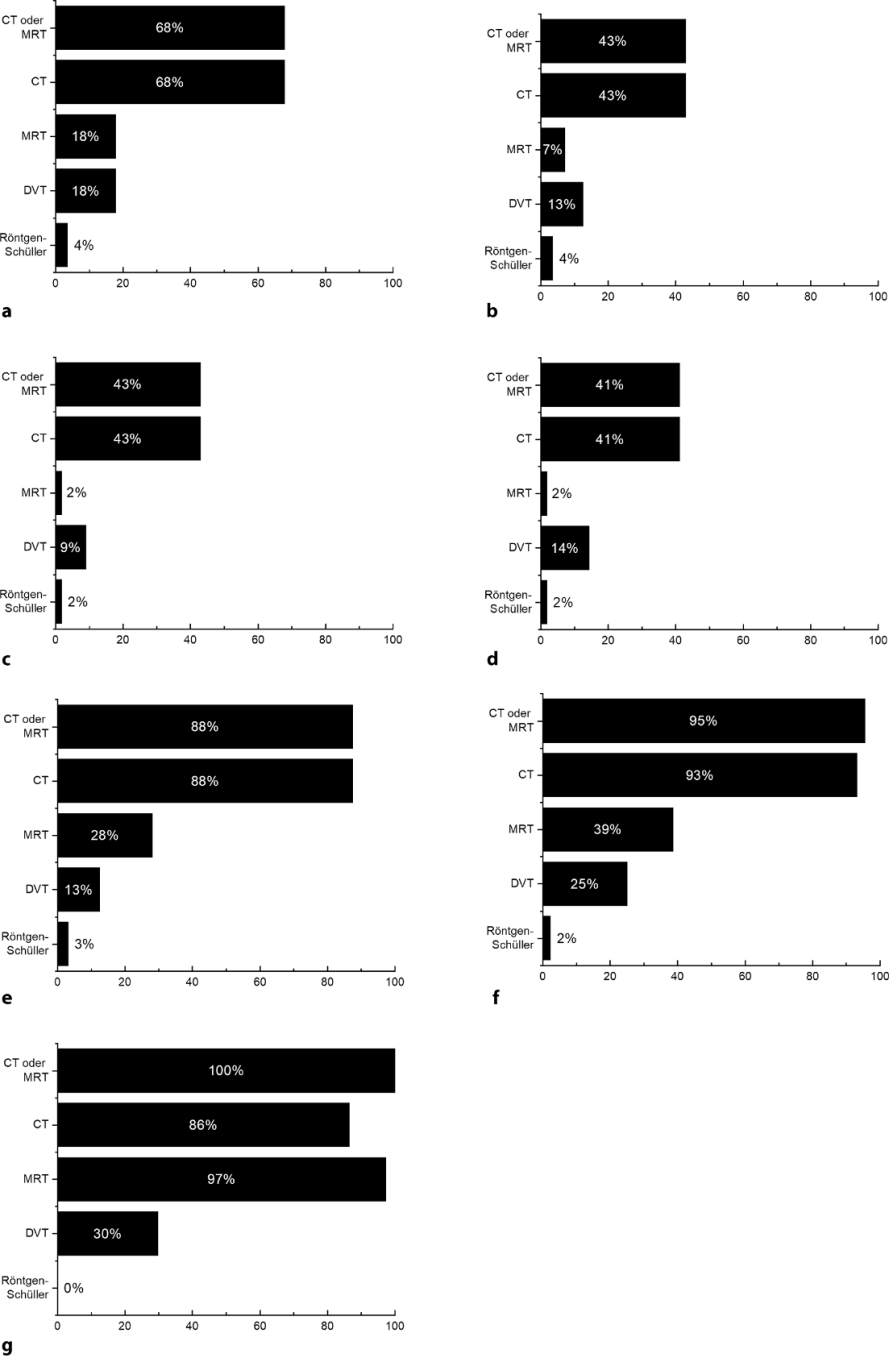
Radiologische Routinediagnostik vor ohrchirurgischen Eingriffen. **a** Sanierende Ohroperationen (*n* = 56), **b** hörverbessernde Operationen (*n* = 56), **c** Stapesplastik (*n* = 56), **d** Gehörgangsstenosen-Operation (*n* = 56), **e** Gehörgangsatresie-Operationen (*n* = 32), **f** implantierbare Hörsysteme (*n* = 44), **g** CI-Operation (*n* = 38). Dargestellt ist jeweils der Anteil der befragten Kliniken, die das jeweilige Diagnostikinstrument routinemäßig vor dem spezifizierten Eingriff einsetzt. *CT* Computertomographie, *MRT* Magnetresonanztomographie, *DVT* digitale Volumentomographie

### Patient-Reported Outcome Measures

Patient-Reported Outcome Measures (PROM) werden routinemäßig in 5 % (*n* = 3) der Kliniken zur präoperativen Evaluation vor Mittelohroperationen eingesetzt. Häufiger werden diese Inventare in der Diagnostik vor einer Hörimplantatversorgung angewendet. In 14 % (*n* = 6) der Kliniken, welche die Versorgung mit implantierbaren Hörsystemen anbieten, werden PROM vor der Implantation zur Diagnostik genutzt. In 34 % (*n* = 13) der Kliniken, in denen Cochleaimplantationen durchgeführt werden, kommen PROM zur präoperativen Evaluation in der Routinediagnostik zur Anwendung.

### Standarddiagnostik vor ausgewählten Ohroperationen

#### Sanierende Ohroperationen

In allen 56 teilnehmenden Kliniken werden sanierende Ohroperationen routinemäßig durchgeführt. Die Tonaudiometrie wird in allen Kliniken präoperativ durchgeführt, in 84 % (*n* = 47) der Kliniken erfolgt präoperativ zusätzliche eine Tympanometrie, in 55 % (*n* = 31) der Kliniken eine Stapediusreflexmessung und in 54 % (*n* = 30) der Kliniken eine Sprachaudiometrie in Ruhe. Weitere subjektive und objektive apparative Hörtestungen werden nur vereinzelt durchgeführt. Eine apparative Vestibularisdiagnostik erfolgt in 32 % (*n* = 18) der Kliniken mittels Kalorik und in 34 % (*n* = 19) mittels Video-Kopfimpulstest. Weitere neurootologische Untersuchungen erfolgen nur sporadisch in den befragten Einrichtungen. In 68 % (*n* = 38) der befragten Kliniken erfolgt eine präoperative Computertomographie (CT).

#### Hörverbessernde Operationen

In allen befragten 56 Kliniken werden hörverbessernde Operationen durchgeführt. Neben der in allen Kliniken obligaten Tonaudiometrie wird vor einer hörverbessernden Operation in der Mehrheit der Kliniken eine Tympanometrie (91 %, *n* = 51), eine Stapediusreflexmessung (63 %, *n* = 35) und eine Sprachaudiometrie in Ruhe (52 %, *n* = 29) durchgeführt. Eine apparative Untersuchung der Gleichgewichtsfunktion erfolgt in nur 16 % (*n* = 9) der Kliniken mittels kalorischer Prüfung und in 21 % (*n* = 12) der Kliniken mittels Video-Kopfimpulstest. In 43 % (*n* = 24) der Kliniken ist die Durchführung einer präoperativen CT obligat.

#### Stapesplastiken

Stapesoperationen werden in allen befragten Kliniken durchgeführt. Zu den routinemäßig in den meisten Kliniken zum Einsatz kommen Testverfahren zählen neben der Tonaudiometrie die Tympanometrie (96 %, *n* = 54), Stapediusreflexmessung (98 %, *n* = 55) und die Sprachaudiometrie in Ruhe (63 %, *n* = 35). Eine neurootologische Diagnostik wird nur in 16 % der Kliniken mittels kalorischer Prüfung (*n* = 9) bzw. in 20 % der befragten Kliniken mittels Video-Kopfimpulstest (*n* = 11) durchgeführt. In 43 % (*n* = 24) der befragten Kliniken wird präoperativ routinemäßig eine CT durchgeführt.

#### Gehörgangsstenosen-Operationen

Gehörgangsstenosen-Operationen werden in allen befragten Kliniken durchgeführt. Zur Routinediagnostik zählen in den meisten Kliniken die Tonaudiometrie (98 %, *n* = 55) und die Tympanometrie (77 % *n* = 43). Eine apparative Funktionskontrolle der Labyrinthorgane erfolgt nur in 5 % (*n* = 3) der Kliniken mittels Kalorik bzw. 16 % (*n* = 9) der Kliniken mittels Video-Kopfimpulstest. In 41 % (*n* = 23) der Kliniken erfolgt eine präoperative Bildgebung mittels CT.

#### Gehörgangsatresie-Operationen

Gehörgangsatresie-Operationen werden in 57 % (*n* = 32) der befragten Kliniken angeboten. Die audiologische Testbatterie umfasst in allen befragten Kliniken eine Tonaudiometrie (100 %, *n* = 32) und eine Sprachaudiometrie in Ruhe (66 % *n* = 21). Weitere objektive und subjektive audiologische Untersuchungen kommen in den befragten Kliniken nur sporadisch zum Einsatz. In 31 % (*n* = 10) dieser Kliniken erfolgt präoperativ ein Video-Kopfimpulstest. Eine CT wird in 88 % (*n* = 28) der Kliniken vor der Atresie-Operation durchgeführt.

#### Implantierbare Hörsysteme

In 79 % (*n* = 44) der an dieser Umfrage teilnehmenden Kliniken werden Patient/-innen mit implantierbaren Hörsystemen versorgt. In der Mehrzahl der Kliniken zählen zur präoperativen audiologischen Testbatterie die Durchführung einer Tonaudiometrie (100 %, *n* = 44), Sprachaudiometrie in Ruhe (98 %, *n* = 43), Tympanometrie (98 %, *n* = 43), Stapediusreflexmessung (82 %, *n* = 36) und die Messung otoakustischer Emissionen (52 %, *n* = 23). Eine neurootologische Diagnostik erfolgt in 41 % (*n* = 18) der Kliniken mittels Kalorik bzw. in 43 % (*n* = 19) der Kliniken mittels Video-Kopfimpulstest. Eine präoperative Bildgebung mittels CT ist in 93 % (*n* = 41) der Kliniken üblich, in 39 % (*n* = 17) erfolgt eine Magnetresonanztomographie (MRT) vor der Implantation.

#### Cochleaimplantat-Operationen

CI-Operationen werden in 68 % (*n* = 38) der befragten Kliniken durchgeführt. Für die CI-Operation zeichnet sich die umfangreichste präoperative Testbatterie in allen Kliniken ab. Die präoperative audiologische Diagnostik beinhaltet mehrheitlich die Durchführung einer Tonaudiometrie (95 %, *n* = 36), Sprachaudiometrie in Ruhe (95 %, *n* = 36) und im Störgeräusch (61 %, *n* = 23), eine Tympanometrie (97 %, *n* = 37), Stapediusreflexmessungen (89 %, *n* = 34) sowie die Registrierung otoakustischer Emissionen (84 %, *n* = 32) und akustisch evozierter Potenziale (89 %, *n* = 34). Zur neurootologischen Diagnostik wird in 76 % (*n* = 29) der Kliniken eine Kalorik und in 79 % (*n* = 30) der Kliniken ein Video-Kopfimpulstest durchgeführt. Eine präoperative Bildgebung mittels CT erfolgt in 84 % (*n* = 32) der befragten Einrichtungen, in 95 % (*n* = 36) der Kliniken wird präoperativ eine MRT durchgeführt.

## Diskussion

Während in den USA bis in die 1990er-Jahre häufiger Umfragen zur Durchführung audiologischer Untersuchungen erfolgt waren, existieren bislang noch keine Untersuchungen, welche audiologische Diagnostik im deutschsprachigen Raum erfassen [24, 25]. Lediglich eine Umfrage im Rahmen eines Workshops der European Academy of Otology and Neuro-Otology 2006 lieferte Hinweise für ein sehr heterogenes Profil der präoperativen Diagnostik und der operativen Durchführung [32].

Einschränkend muss für die aktuell erfolgte Umfrage eingeräumt werden, dass sich vorzugsweise Kliniken mit einer hohen Expertise in der Ohrchirurgie beteiligten. Dies lässt sich anhand der Operations-, ärztlich tätigen Mitarbeiter- und Funktionsdienstmitarbeiteranzahl sowie der Mitarbeiterqualifikationen und des Versorgungsspektrums festmachen.

Die Bedeutung einer hochwertigen audiologischen Diagnostik im deutschsprachigen Raum schlägt sich beispielsweise in der Herausbildung zertifizierter Audiologischer Zentren wieder, welche hinsichtlich einer integrierten Diagnostik und Therapie von Hörstörungen definierte Kriterien einer interprofessionellen personellen, technischen und räumlichen Ausstattung erfüllen und über definierte qualitätsgesicherte Prozesse verfügen [7]. Trotz einheitlicher Zertifizierungsregularien obliegt es bislang jedem Zentrum selbst, Testbatterien für unterschiedliche Krankheitsbilder und Versorgungswege zu erarbeiten. Die Erarbeitung entsprechender krankheitsbildabhängiger Diagnostiksets muss dabei die gängige Lehrmeinung, vorhandene Leitlinien, medikolegale Aspekte und Anforderungen der Kostenträger berücksichtigen und gleichzeitig den ökonomischen Randbedingungen entsprechen. Zudem besteht der individuelle Anspruch der Operateur:innen, anhand der Diagnostik nicht nur eine Diagnose oder Interventionsindikation zu stellen, sondern auch Parameter zur Erfolgskontrolle und zur Erfolgsprognose zu erheben. Insbesondere letzterem Aspekt kommt bei Elektiveingriffen oder Eingriffen mit Alternativoptionen ein hoher Stellenwert zu. Dies zeigt sich in der vorliegenden Umfrage insbesondere für hörverbessernde Eingriffe, inkl. der Stapeschirurgie und der Versorgung mit implantierbaren Hörsystemen. Der audiologische Fokus liegt hier neben der obligatorischen Reintonaudiometrie auf der Durchführung der Sprachaudiometrie. Das Tonaudiogramm stellt nach wie vor den Goldstandard in der Diagnostik der Hörstörungen dar. Aus dem Verlauf von Luft- und Knochenleitungsschwellen lassen sich einerseits Art und Grad der Hörstörung bestimmen. Andererseits lassen typische Kurvenverläufe Rückschlüsse auf die Ursache des Hörverlusts zu und unterstützen die Planung des chirurgischen Vorgehens sowie die Patient/-innenberatung. Als Goldstandard ist dieses Verfahren einerseits in den verfügbaren otologischen Leitlinien implementiert [9–12]. Andererseits ist dieses Verfahren seit jeher integraler Bestandteil der in der Fachliteratur dargelegten Diagnostikbatterien für otologische Krankheitsbilder und unverzichtbarer Bestandteil bei Begutachtungen [1, 14, 16, 33].

Insbesondere bei der Versorgung mit implantierbaren Hörsystemen spielt an dieser Stelle auch die Nachweispflicht gegenüber den Kostenträgern eine entscheidende Rolle. Während in der Leitlinie „Implantierbare Hörgeräte“ und „Cochlea-Implantat Versorgung“ die Sprachaudiometrie, insbesondere die Hörsystemüberprüfung, explizit implementiert ist, ist deren Durchführung in der Leitlinie „Cholesteatom“ nicht und in der Leitlinie „chronisch mesotympanale Otitis media“ als fakultatives Testverfahren hinterlegt [9–12]. Umso erstaunlicher zeigt sich in der vorliegenden Umfrage eine Implementierung dieses zeitaufwendigeren Testverfahrens vor sanierenden Ohroperationen in mehr als der Hälfte der befragten Kliniken. Bei den Elektiveingriffen zur Hörverbesserung ist die Sprachaudiometrie in nur 50 %, vor Stapesplastiken immerhin in 70 % der Kliniken bereits Routine. Da das primäre Ziel dieser Eingriffe die Verbesserung des Sprachverstehens ist, erscheint es verwunderlich, dass das Sprachverstehen selbst von 50 % bzw. 30 % der teilnehmenden Kliniken nicht als Zielparamater erfasst wird. Anhand der Bestimmung des prä- und postoperativen Sprachverstehens lässt sich einerseits der Erfolg hörverbessernder Operationen dokumentieren. Andererseits unterstützen sprachaudiometrische Untersuchungen die Beratung der Patient/-innen vor der Operation hinsichtlich eines realistischen Operationsziels und sind insbesondere bei Patient/-innen mit kombinierter Schwerhörigkeit zur Abgrenzung gegenüber der Hörimplantatversorgung nötig. Auch mit Hinblick auf die zunehmende Bedeutung medikolegaler Fragestellungen erscheint der geringe Anteil von Kliniken, welche vor konventionellen Ohroperationen sprachaudiometrische Untersuchungen durchführen, überraschend gering. Dies deckt sich jedoch mit der aktuellen wissenschaftlichen Praxis. In einer Bewertung der Publikationsqualität von Originalarbeiten, welche Ergebnisse nach hörverbessernden Operationen bewerteten, wurden sprachaudiometrische Ergebnisse nur in 4 % (7 von 169 Publikationen) dargestellt [21]. Die in der aktuellen Literatur oftmals vorgenommene Fokussierung auf den Vergleich der prä- und postoperativen Schallleitungskomponente muss an dieser Stelle als methodisch ungenügend eingestuft werden, da somit eine ggf. auch nur frequenzspezifisch auftretende Änderung der Knochenleitungshörkurve zu einer Verzerrung der Bewertung der Operationserfolgs führen kann [34].

In der hier vorliegenden Umfrage fiel zudem auf, dass in 2 % der befragten Kliniken vor der Versorgung mit implantierbaren Hörgeräten keine Sprachaudiometrie sowie in 5 % der teilnehmenden Kliniken vermeintlich vor einer CI-Operation keine Ton- und Sprachaudiometrie durchgeführt wird. Da dies ein aus Sicht der Autoren nicht nachzuvollziehendes Vorgehen darstellen würde, ist hier am ehesten ein Eingabefehler anzunehmen, da innerhalb des Online-Fragebogens keine Plausibilitätskontrolle integriert war.

In der Umfrage zeichnet sich eine Inhomogenität der einer Sprachaudiometrie entnommenen Zielparameter ab. Dies deckt sich mit der Analyse der derzeitigen Publikationsqualität [27], aber auch mit den Ergebnissen einer bereits Mitte der 1990er-Jahre durchgeführten amerikanischen Umfrage unter Audiolog/-innen [24, 25]. Bislang existiert auch in Deutschland kein dokumentierter Standard zur Erfassung sprachaudiometrischer Parameter vor und nach ohrchirurgischen Eingriffen [28]. Die in den Empfehlungen der amerikanischen Fachgesellschaft getroffene Festlegung zur Bestimmung der Sprachverständlichkeit bei 40 dB über der Sprachverständlichkeitsschwelle oder maximal komfortablem Sprachschallpegel [17] wurde für die Mittelohrchirurgie in einer audiologischen Bewertung als methodisch unzureichend eingestuft [27, 28]. Bei Patient/-innen mit mittel- bis hochgradiger Schwerhörigkeit ist eine Messung des Sprachverstehens bei 40 dB über SRT prä- und/oder postoperativ nicht möglich, da die Pegelgrenzen eines Audiometers rasch erreicht sind. Besonders bei Patient/-innen mit Schallleitungsschwerhörigkeiten, welche präoperativ oft ein maximales Einsilberverstehen von 100 % aufweisen, kann keine Verbesserung des bei 40 dB über der SRT ermittelten Sprachverstehens nach der Operation erwartet werden. Außerdem kann die Bestimmung des Sprachverstehens bei 40 dB über der SRT zu einer Fehlinterpretation des Behandlungserfolgs führen: Bei einigen Patient/-innen kann eine Hörverbesserung in den tiefen Frequenzen durch die Operation zu einer Verschiebung des SRT-basierten Pegels führen und somit das gemessene Sprachverstehen methodenbedingt beeinflussen [28]. Im deutschsprachigen Raum wird dieses Vorgehen in keiner der an der Umfrage teilnehmenden Kliniken gewählt. Auch bei der Betrachtung der internationalen Studienlage zeigte sich, dass die Messung bei 40 dB über der SRT sich bislang nicht durchgesetzt hat und in weniger als 10 % der publizierten Untersuchungen zur Hörverbesserung nach Operationen eingesetzt wird [27]. Die Wahl der Methode bei der Durchführung der Sprachaudiometrie beeinflusst an dieser Stelle stark die Wertigkeit dieser Diagnostikmethode in der Beurteilung der Hörveränderung. In einer klinischen Untersuchung hatte sich unlängst gezeigt, dass die Messung bei einem festen Sprachschallpegel die verlässlichsten Ergebnisse lieferte [28]. Die aktuelle Umfrage zeigt, dass in 41 % der Kliniken eine Messung bei festem Pegel erfolgt. Dieser Pegel differierte jedoch klinikübergreifend, wobei in den meisten Kliniken die Messung bei 65 dB SPL (34 %) und 80 dB SPL (29 %) durchgeführt wird. In einer Analyse der Publikationsqualität von klinischen Studien zu Hörergebnissen nach Ohr-Operationen hatte sich gezeigt, dass die Messung des Sprachverstehens bei festem Pegel den höchsten Stellenwert hat, wobei sich hier auch eine heterogene Pegelauswahl abzeichnete bzw. die Dokumentation des genutzten Sprachschallpegels oft mangelhaft ist [27]. Im deutschsprachigen Raum hat derzeit die Messung mit Einsilbern bei einem festen Pegel von 65 dB SPL die größte Bedeutung [26–28, 31, 38]. Die Messung bei 65 dB SPL mit Einsilbern ist aktuell im Weißbuch CI, als Dokumentationsanforderung im CI-Register, in der Leitlinie zur Cochleaimplantatversorgung sowie in einem Konsensusstatement zur audiologischen Berichtserstattung nach der Versorgung mit aktiven Mittelohrimplantaten hinterlegt [8, 12, 23]. Im deutschsprachigen Raum existiert bislang jedoch noch kein dokumentierter Standard für die Auswahl sprachaudiometrischer Zielparameter nach konventionellen Ohroperationen [27, 28]. Auch die Messung des maximalen Sprachverstehens hat deutschlandweit weiterhin einen festen Stellenwert, eignet sich jedoch vordergründig zur Indikationsstellung für die Versorgung mit implantierbaren Hörgeräten oder Cochleaimplantaten. Weniger eignet sich diese Zielparameter der Sprachaudiometrie zur Bewertung der Hörverbesserung nach konventionellen Mittelohroperationen [28]. Diese Ergebnisse unterstreichen, dass nicht nur eine Vereinheitlichung der Methodenauswahl für Testbatterien, sondern auch eine Standardisierung dieser Methoden anzustreben ist.

In den früheren Jahren erfolgte präoperativ oftmals aus medikolegalen Gründen noch eine Röntgenuntersuchung des Felsenbeins, insbesondere die Spezialaufnahme nach Schüller. Während 2006 in einer europäischen Umfrage noch 20 % der Operateure angaben, vor einer Cholesteatomoperation oder Tympanoplastik eine Röntgenuntersuchung durchzuführen [32], zeigte sich in der aktuellen Umfrage, dass diese in nahezu allen befragten Kliniken in der präoperativen Diagnostik keinen Stellenwert mehr hat. Dies deckt sich mit der gängigen Fachliteratur, in der diese zur präoperativen Diagnostik nicht einmal mehr erwähnt wird [18, 19].

Während Einigkeit über die fehlende Aussagekraft der Röntgenuntersuchung herrscht, besteht noch kein Konsensus hinsichtlich der Notwendigkeit der Durchführung einer CT des Felsenbeins vor Mittelohreingriffen. Im europäischen Vergleich zeichnet sich für den deutschsprachigen Raum noch eine Zurückhaltung in der Beurteilung deren Notwendigkeit ab. In einer europäischen Umfrage hatten 2006 93 % der Chirurgen angegeben eine CT vor Cholesteatom-Operationen durchzuführen [32], während nur 60 % der an der aktuellen Umfrage beteiligten Kliniken diese in der Routinediagnostik einsetzen. Auch hinsichtlich der Wertigkeit der Bildgebung vor Stapesplastiken zeigt sich im deutschsprachigen Raum noch keine Einigkeit. Lediglich in 30 % der befragten Kliniken erfolgt eine CT routinemäßig, im europäischen Raum in 48 % der Fälle. Vermutlich begrenzt sich die Indikationsstellung zur CT vor Stapesplastiken auf Unsicherheiten bei der Diagnosestellung und Revisionsfälle, wobei nicht vernachlässigt werden sollte, dass in bis zu 30 % der CT-Bildgebungen bei intaktem Trommelfell Auffälligkeiten wie Kettenunterbrechung, Dysplasien, Hammerkopffixation, Malformation, Bogengangdehiszenzen und Innenohrfehlbildungen identifiziert werden können [13].

Die aktuellen Daten bekräftigen, dass an dieser Stelle die nationale Erarbeitung eines Konsensus zur Indikationsstellung für eine Bildgebung angestrebt werden muss. Klare Indikationskriterien können an dieser Stelle auch zur einheitlichen Bearbeitung gutachterlicher Fragestellungen beitragen. Preuss et al. zeigten, dass Chirurg/-innen vordergründig die Indikation zur Durchführung einer CT bei Revisionseingriffen und schwerwiegenden Komplikationen sehen [32].

Eine geringe Bedeutung kommt vor konventionellen ohrchirurgischen Eingriffen einer apparativen Untersuchung des Gleichgewichtssystems zu. In teilweise weniger als einem Fünftel der befragten Kliniken wird vor sanierenden Ohroperationen, hörverbessernden Operationen inkl. Stapesplastik oder Gehörgangsstenosenoperationen eine Untersuchung der Labyrinthfunktion (Kalorik oder Video-Kopfimpulstest) routinemäßig durchgeführt. In diesem Bereich zeichnet sich damit eine Rationalisierung der Diagnostik ab, da die Diagnostik des Gleichgewichtssystems, ausgenommen Patient/-innen mit Komplikationen, die chirurgische Therapie weder in der Indikationsstellung noch im Vorgehen beeinflusst. Hingegen kann entsprechend der im Rahmen der Umfrage ermittelten Angaben eine umfassende Diagnostik des Gleichgewichtsorgans vor Versorgung mit Cochleaimplantaten als obligat angesehen werden. Die Implementierung der neurootologischen Diagnostik im CI-Versorgungsprozess spiegelt dabei auch das leitlinienkonforme Vorgehen bzw. die Umsetzungen der Empfehlungen des Weißbuches CI-Versorgung wider, in denen diese Diagnostik klar hinterlegt ist [8, 12]. In den Leitlinien „Cholesteatom“, „Chronisch mesotympanale Otitis media“ und „Implantierbare Hörgeräte“ wird bislang zur Notwendigkeit der Durchführung einer neurootologischen Diagnostik keine Stellung bezogen [9–11].

Generell zeigt sich, dass Kliniken bei konkreten Vorgaben zur Diagnostik in Leitlinien diese auch in den eigenen Testbatterien umsetzen. An dieser Stelle ist der CI-Versorgungsprozess hervorzuheben. In der aktuellen Leitlinie und im Weißbuch werden für diesen Prozess klare Durchführungsbestimmungen geliefert. Mittlerweile wird ein Weißbuch-konformes Vorgehen auch durch die Pflicht zur Zertifizierung als CI-versorgende Einrichtung sowie Datenweitergabe an ein CI-Register unterstützt. Möglicherweise ergibt sich in der hier vorliegenden Untersuchung ein Bias, da 68 % der teilnehmenden Klinik CI-Operationen durchführen und durch die Notwendigkeit der Zertifizierung als CI-versorgende Einrichtung klar definierte fachliche Anforderungen erfüllt werden müssen [36]. Die Einhaltung von Leitlinien und Behandlungsstandard durch Kliniken war bislang für externe Betrachter inkl. Patient/-innen nicht möglich. Insgesamt tragen Zertifizierungssysteme dazu bei, Standards für die Prozess‑, Struktur- und Ergebnisqualität zu erzielen und aufrechtzuerhalten. Auch die Zertifizierung als Audiologisches Zentrum (Deutsche Gesellschaft für Audiologie) trägt mittlerweile zur Qualitätssicherung bei. Eine bislang überschaubare Anzahl der zertifizierten Zentren steht an dieser Stelle jedoch einer größeren Anzahl von Kliniken gegenüber, welche ohrchirurgische Eingriffe durchführen [7].

Die auch in der CI-Leitlinie geforderte Erfassung von Patient-Reported Outcome Measures (PROM) ist nur in einer Minderheit der Kliniken (34 %) in den Testbatterien vor der CI-Operation implementiert. Vor Mittelohreigriffen werden PROM lediglich in 5 % der befragten Kliniken genutzt. Insgesamt zeigen die im Rahmen der Umfrage erhobenen Ergebnisse, dass PROM in der Otologie in der Routineversorgung nur einen geringen Stellenwert aufweisen. Obwohl seit den 2000er-Jahren auch in der Otologie das Interesse an der Bereitstellung krankheitsspezifischer PROM wächst und diese auch zunehmend in klinischen Studien analysiert und mit Funktionsparametern abgeglichen werden, steht die Implementierung im Gegensatz zu anderen Fachgebieten noch am Anfang. Momentan existieren neben den bekannten generischen Messinstrumenten nahezu für jedes otologische Krankheitsbild validierte deutschsprachige Messinstrumente [2, 3, 20, 30]. Schlussendlich sollte der zunehmenden Bedeutung von PROM zur Qualitätsbeschreibung medizinischer Versorgungswege Rechnung getragen werden und deren Integration in die klinische Routine weiter vorangetrieben werden.

Gerade eine klinikübergreifende Auswertung von in zentralen Datenbanken erfassten Zielparametern liefern zum einen Kenntnisse über die generelle Versorgungsqualität, erlauben ein Benchmarking und können zur Bewertung von Therapie in größeren Patient/-innenpopulationen beitragen. Ein hohes Interesse der Erfassung der klinikeigenen Versorgungsqualität spiegelt sich auch in der mittlerweile breiten Verfügbarkeit von Datenbanksystemen zur Erfassung der Ergebnisse nach Ohroperationen wider. Aktuell nutzen 45 % der Kliniken eine entsprechende Datenbank. Um eine klinikübergreifende Auswertung durchzuführen, bedarf es einer Standardisierung der Zielparameter. Da im Rahmen klinischer Studien nahezu regelhaft auch auf Routineparameter aus der Krankenversorgung zurückgegriffen wird, lässt eine Standardisierung der Diagnostikbatterien auch eine Verbesserung der Studienqualität und vor allem der Vergleichbarkeit von in klinischen Studien erhobenen Ergebnissen in der Ohrchirurgie erwarten.

Innerhalb dieser Umfrage wurden Diagnostikbatterien für Erwachsene vor Ohr-Operationen erfasst. Für die Diagnostik der kindlichen Schwerhörigkeit vor Parazentese und Paukendrainage, ggf. in Kombination mit einer Adenotomie oder Tonsillektomie bzw. Tonsillotomie ist ebenfalls bislang keine standardisierte Testbatterie definiert. Auch hier ergibt sich für die beteiligten Fachgesellschaften ein Auftrag zur Definition notwendiger Untersuchungen und deren Integration in zukünftige Leitlinien.

Die hier erhobenen Daten beruhen auf der Eigeneinschätzung der an der Umfrage teilnehmenden Kliniken. Eine unabhängige Prüfung der Informationen auf Richtigkeit und Vollständigkeit der gemachten Angaben konnte nicht erfolgen. Abweichungen der Angaben sind demnach möglich. Dennoch liefern die Ergebnisse dieser Umfrage durch die Analyse der aktuellen Diagnostik im deutschsprachigen Raum eine weitere Diskussionsgrundlage zur Erarbeitung eines Diagnostikstandards zur Integration in entsprechende Leitlinien bzw. für eine Konsensbildung innerhalb der Fachgesellschaft.

## Fazit für die Praxis

- Die vorliegende Arbeit ist eine erste Standortbestimmung zur Anwendung präoperativer Diagnostikverfahren in der Ohrchirurgie im deutschsprachigen Raum. Klinikübergreifend zeichnet sich eine Inhomogenität der präoperativen Testbatterien ab.
- Die in den verfügbaren Leitlinien, z. B. in der Leitlinie „Cochlea-Implantat Versorgung“ standardisierten Diagnostikverfahren werden im klinischen Alltag in der Routinediagnostik berücksichtigt.
- Bei der Erarbeitung und Überarbeitung weiterer Leitlinien bzw. bei der Erstellung entsprechender Konsensuspapiere der Fachgesellschaften und Arbeitsgemeinschaften kann auf die hier präsentierten Daten zurückgegriffen werden, um rationale, standardisierte Diagnostikbatterien für weitere ohrchirurgische Eingriffe zu etablieren.

## Data Availability

Rohdaten können auf Anfrage zugänglich gemacht werden.
